# The herbal drug, Bu-Zhong-Yi-Qi-Tang, for the treatment of atopic dermatitis: Protocol for a systematic review

**DOI:** 10.1097/MD.0000000000013938

**Published:** 2019-01-04

**Authors:** Mi-Kyung Jeong, Young-Eun Kim, Anna Kim, Jeeyoun Jung, Mi Ju Son

**Affiliations:** a Clinical Medicine Division; b Future Medicine Division, Korea Institute of Oriental Medicine, Yuseongdae-ro, Yuseong-gu, Daejeon, Republic of Korea.

**Keywords:** atopic dermatitis, Bojungikki-tang, Bu-Zhong-Yi-Qi-Tang, eczema, herbal medicine, Hochu-ekki-to, systematic review

## Abstract

**Introduction:**

Bu-Zhong-Yi-Qi-Tang (BZYQT) is an herbal drug that is widely used to treat various diseases, including gastrointestinal diseases, allergic rhinitis, and atopic dermatitis (AD) in East Asian countries. BZYQT has been shown to have anti-allergic, anti-inflammatory, and immunoregulatory properties in experimental studies, and there is substantial clinical evidence of its effect on AD. This review will systematically assess the evidence of BZYQT for the treatment of AD.

**Methods/design:**

Eleven databases will be searched from their inception without language restriction. Randomized controlled trials that examined BZYQT or modified BZYQT for AD will be included. The selection of the studies, data abstraction, and validations will be performed independently by 2 researchers. The methodological qualities of the randomized controlled trials will be assessed using the Cochrane Collaboration tool for assessing the risk of bias.

**Ethics and dissemination:**

This systematic review will be published in a peer-reviewed journal and will also be disseminated electronically or in print. It will be useful to inform and guide healthcare practitioners.

**Trial register number:**

CRD42018105173.

## Abstract 2

Supplemental Digital Content is available in the text

## 1 Introduction

Atopic dermatitis (AD) is a chronically relapsing inflammatory skin disease that affects approximately 15% to 30% of children and 2% to 10% of adults.^[1]^ The onset of AD usually occurs in childhood, with 60% of patients experiencing an eruption in the first year.^[2]^ Major symptoms of AD are characterized by itching or pruritus and chronic recurrent inflammation in a manner that typically shows age-related morphology and distribution. Although clinical features differ with each age group, eczematous rashes, loss of sleep, and impact on the psychosocial wellbeing of patients are common in all age groups.^[3,4]^ The causes of AD are still uncertain; however, interactions among genetic factors are a probable cause, which are associated with skin barrier function and altered cutaneous immune responses, as well as environmental factors, such as early-life gut bacteria, humidity, microbes, and allergens.^[5,6]^

Topical administration of corticosteroids or calcineurin inhibitors are regarded as a standard first-line treatment for AD.^[7]^ However, long-term application of topical corticosteroids has been associated with adverse cutaneous effects including atrophy, rebound flares, and increased percutaneous absorption with the potential for adverse systemic effects.^[8]^ In addition, the use of topical calcineurin inhibitors has been associated as a potential risk factor for cancer. Likewise, systemic medications including oral corticosteroids or immunosuppressive drugs, which are used for refractory AD, have also been reported to have adverse systemic effects such as hepatosplenic T-cell lymphomas.^[9–11]^ Due to these adverse effects of conventional medical treatment, many AD patients prefer herbal medicine in the form of complementary and alternative medicine for improving AD symptoms.^[12]^

Bu-Zhong-Yi-Qi-Tang (BZYQT), also known as “Hochu-ekki-to” in Japan, and “Bojungikki-tang” in Korea, is an herbal drug extensively used for treatment of various diseases, such as gastrointestinal diseases, allergic rhinitis, and AD, in East Asian countries. It has been reported that BZYQT has anti-allergic properties through suppression of serum immunoglobulin E (IgE) levels and eosinophil infiltration, and controlling T-helper 1/T-helper 2 (Th1/Th2) balance.^[13–17]^ Moreover, BZYQT also has immunomodulatory effects that prevent serum IgE level increase and correct the Th1/Th2 balance skewed to Th2 in atopic NC/Nga mice.^[18,19]^ In addition, studies have reported on the protective effect of BZYQT against oxidative skin stress.^[20]^ Likewise, many studies have been reported BZYQT to be effective in treating AD patients.^[21–24]^

However, there is no evident critical appraisal, such as a systematic review or meta-analysis of the potential benefits and harms of BZYQT on AD. Therefore, in this present study, we aim to conduct a systematic review of randomized controlled trials (RCTs) to assess the evidence on the effectiveness of BZYQT for treating AD.

## 2 Methods

### 2.1 Study registration and ethics

The protocol for this systematic review has been registered in PROSPERO (https://www.crd.york.ac.uk/PROSPERO) under the number CRD42018105173 and was written in accordance with the preferred reporting items for systematic reviews and meta-analysis protocol (PRISMA-P) guidelines.^[25]^ This systematic review is not necessary for ethical approval because individual patient data cannot be identified.

### 2.2 Type of studies

All RCTs and quasi-RCTs will be included and any trials without parallel comparisons or control groups will be excluded.

### 2.3 Type of participants

This study will include AD patients of any age with the following criteria: diagnosis of AD (or atopic eczema) using clinical diagnosis or validated diagnostic criteria.

### 2.4 Type of interventions

In this study, both BZYQT and modified BZYQT will be included. BZYQT is composed of 8 medical plants, Astragali radix, Atractylodis Rhizoma Alba, Ginseng radix, Angelicae Gigantis Radix, Citri Unshius Pericarpium, Glycyrrhizae Radix et Rhizoma, Cimicifugae Rhizoma, and Bupleuri radix. Modified BZYQT is added or removed medicinal plants according to pattern identification, or syndrome differentiation, resulting in nearly the same actions as the original BZYQT. If orally administered, any formulation of BZYQT will be included. There is no limitation on the number of herbs, dosage, or duration of treatment.

### 2.5 Type of comparisons

All types of controls such as placebo, conventional treatment, or no treatment will be included. Trials in which BZYQT was used as the only treatment or as an adjunct to other treatments, as well as those in which the control group received the same treatment as the intervention group will be included.

### 2.6 Outcome measures

#### 2.6.1 Primary outcomes

1. Symptom severity assessment tools that evaluate the extent and intensity of skin lesions, such as the SCORing atopic dermatitis index (SCORAD) and eczema area severity index (EASI).
2. Total effectiveness rate.
3. Percentage of trial participants with the sum of “recovery” and “significant improvement” reported by the trial investigators itching visual analogue scale.

### 2.7 Secondary outcomes

1. Adverse effects measured by any relevant incidence and duration of any side effects.
2. Validated quality of life assessment tools such as the dermatology life quality index (DLQI).
3. Concurrent therapies: the dosage of topical agents expressed as the total equivalent amount and assessment of the add-on effect of BZYQT combined with topical agents.

### 2.8 Data sources

We will search for trials from the following electronic databases: MEDLINE, EMBASE, the Cochrane Central Register of Controlled Trials (CENTRAL), AMED, and the Cumulative Index to Nursing and Allied Health Literature (CINAHL). We will also search 4 Korean medical databases (Oriental Medicine Advanced Searching Integrated System [OASIS], Korean studies Information Service System [KISS], National Digital Science Library [NDSL], and KoreaMed), 1 Chinese database (China Network Knowledge Infrastructure [CNKI]), and 1 Japanese Database (CiNii). For ongoing trials, trials will be searched on the metaRegister of Controlled Trials (mRCT; http://www.controlled-trials.com/mrct/), Clinical trials.gov (http://www.ClinicalTrials.gov), and the WHO International Clinical Trials Registry Platform (ICTRP) (http://apps.who.int/trialsearch/). We will also check the reference lists of reviews and the retrieved articles for additional studies. The search strategies that will be applied to the MEDLINE and CNKI are presented in Appendix 1. Similar search strategies will be applied for all databases. All bibliographic information and articles will be managed using EndNote (X8.2; Clarivate Analytics, Philadelphia).

### 2.9 Selection of studies

Three reviewers will review and screen the titles and abstracts to identify eligible trials based on the inclusion criteria. Disagreements will be resolved by discussion, and if required, by the arbiter. Details of the study selection procedure are summarized in a PRISMA-compliant flow diagram.^[26]^

### 2.10 Data extraction

We will extract the following information from the included systematic reviews: bibliographic information (e.g., author, publication date, and country), population demographics and setting (e.g., age, sex, and sample size), type of intervention (e.g., dosage, regimen, administration method, and herbal composition of prescription), outcome measures, and adverse events. Two authors will perform data extraction using a predefined data extraction form to record descriptive characteristics of the included reviews. Disagreements will be resolved by discussion among all of the authors, and another author will act as an arbiter for unresolved disagreements. Extracted data will be presented as included study summary table.

### 2.11 Quality assessment

The risk of bias will be assessed in accordance with criteria from the Cochrane Handbook version 5.2.0, which includes random sequence generation, allocation concealment, blinding of participants and personnel, blinding of outcome assessment, incomplete outcome data, selective reporting, and other sources of bias.^[27]^ The quality of each trial will be categorized on the basis of low/unclear/high risk of bias. We will use the Grading of Recommendations Assessment, Development and Evaluation (GRADE) approach to assess confidence in estimates of effect.^[28]^

### 2.12 Data synthesis

Differences between the intervention and control groups will be assessed in this study. For continuous data, we will use the mean difference (MD) with 95% confidence interval (CI) to measure the treatment effects. We will convert other forms of data into MD. For outcome variables on different scales, we will use the standard MD with 95% CI. For dichotomous data, we will present treatment effect as a relative risk (RR) with 95% CI; other binary data will be converted into RR. All statistical analyses will be conducted using Cochrane Collaboration's software program, Review Manager (RevMan), version 5.3 for Windows (Copenhagen, Denmark, The Nordic Cochrane Centre, the Cochrane Collaboration, 2014). If appropriate, we will pool data across studies for a meta-analysis using fixed-effects and random-effects models with 95% CI.

### 2.13 Unit of analysis issue

For crossover trials, the data from the first treatment period will be used. For trials in which more than 1 control group is assessed, the primary analysis will combine the data from each control group. Subgroup analyses of the control groups will be conducted. Each patient will be counted only once in the analyses.

### 2.14 Dealing with missing data

If we find that data is missing when we include the data, we will consider the reason for the loss of data. And then, we will contact the corresponding authors to acquire and verify data wherever possible. If it is not possible to do this, we will only analyze the available data.

### 2.15 Assessment of heterogeneity

If meta-analysis is possible, the I^2^ tests will be used to evaluate the heterogeneity of the included studies, where I^2^ > 50 will indicate high heterogeneity. In the case of heterogeneity, we will conduct subgroup analyses to explore the possible causes.^[29]^ We will use the random or fixed-effects model in this study for meta-analysis according to the data analysis.

### 2.16 Assessment of reporting biases

If a sufficient number of included studies (at least 10 trials) are available, we will use funnel plots to detect reporting bias. However, as funnel plot asymmetry is not identical to publication bias, we will attempt to distinguish the possible reasons for any asymmetries, such as small-study effects, poor methodological qualities, and true heterogeneities.^[30,31]^

## 3 Discussion

Recently, there has been an increase in the use of complementary and alternative medicine for treating AD worldwide.^[12,32]^ A Cochrane systematic review evaluating the effects of herbal medicine on AD showed that oral application of herbal medicine was superior to conventional medicine in total effectiveness rate (RR 1.43, 95% CI 1.27–1.61) and itching visual analogue scale (standard MD, 95% CI 1.43–0.22).^[33]^ Furthermore, herbal medicine treatment for AD was recommended in the Korean Medicine Clinical Practice Guideline as grade B, which states that herbal medicine must be considered before conventional medicine based on the clinician's opinion.^[34]^

BZYQT is a widely used herbal medicine in Asian countries, such as China, Japan, and Korea. BZYQT, which is referred to as a decoction to tonify the middle and augment the Qi formula, was first recorded in Dong Yuan Ten Medical Books, a medical text written by Li Dong-yuan in the year 1249.^[35]^ It is known as a herbal treatment for gastrointestinal diseases, cancer, and chronic fatigue syndrome associated with the syndrome of “sinking of qi due to spleen deficiency,” a concept of traditional medicine.^[36–38]^ Furthermore, the Chinese Clinical Guidelines for AD reported deficiency of the spleen with accumulation of dampness to be the most common syndrome of childhood AD, and recommended using BZYQT for the treatment of pediatric AD.^[39,40]^ The pathogenesis of AD is complex and multifactorial; however, the systemic immune response plays a major role in pathogenesis in AD patients. Most patients with AD have eosinophilia and increased serum IgE concentrations.^[5,6]^ Many studies using various experimental models have shown that BZYQT has immunoregulatory effects.^[13–19]^ These properties are also beneficial for alleviating AD symptoms in clinical practice.

Thus, in this study, we will investigate the clinical evidence related to the effectiveness of BZYQT for treating AD. All BZYQT formulations, such as decoctions, pills, capsules, and tablets, will be considered in this study. Furthermore, trials for which the assessed BZYQT involved a different ratio of ingredients or missing herbs compared to the original BZYQT will be included if similar to the original BZYQT. Detailed information about variants of BZYQT will be provided, including their composition, ingredient ratio, type of formulation, and prescription based on the pattern identification. Thus, this systematic review will provide evidence for the use of BZYQT in the treatment of AD. Moreover, as our systematic review will involve an unbiased search of various databases including Asian databases without language restrictions, readers will obtain the opportunity to access studies originally published in East Asian languages that they would otherwise be unable to read.

## Author contributions

M.K.J. and M.J.S. conceived the study, developed the study criteria, and wrote the protocol. M.K.J. and Y.E.K. conducted the preliminary search. A.K. examined the relevance of the protocol in clinical practice. M.J.S. and J.J. revised the manuscript. All authors have read and approved the final manuscript.

**Conceptualization:** Jeeyoun Jung.

**Investigation:** Mi-Kyung Jeong, Young-Eun Kim, Anna Kim, Mi Ju Son.

**Methodology:** Mi Ju Son.

**Supervision:** Jeeyoun Jung.

**Writing – original draft:** Mi-Kyung Jeong.

**Writing – review & editing:** Mi Ju Son, Jeeyoun Jung.

Mi Ju Son orcid: 0000-0003-1701-9122.

## Supplementary Material

Supplemental Digital Content

## References

[1] Bieber T Atopic dermatitis. N Engl J Med 2008;358:1483–94. 18385500 10.1056/NEJMra074081

[2] Illi S von Mutius E Lau S The natural course of atopic dermatitis from birth to age 7 years and the association with asthma. J Allergy Clin Immunol 2004;113:925–31. 15131576 10.1016/j.jaci.2004.01.778

[3] Eichenfield LF Tom WL Chamlin SL Guidelines of care for the management of atopic dermatitis: section 1. Diagnosis and assessment of atopic dermatitis. J Am Acad Dermatol 2014;70:338–51. 24290431 10.1016/j.jaad.2013.10.010 PMC4410183

[4] Baron SE Cohen SN Archer CB British Association of Dermatologists and Royal College of General Practitioners. Guidance on the diagnosis and clinical management of atopic eczema. Clin Exp Dermatol 2012;37 Suppl 1:7–12. 22486763 10.1111/j.1365-2230.2012.04336.x

[5] Lyons JJ Milner JD Stone KD Atopic dermatitis in children: clinical features, pathophysiology, and treatment. Immunol Allergy Clin North Am 2015;35:161–83. 25459583 10.1016/j.iac.2014.09.008 PMC4254569

[6] Leung DY Bieber T Atopic dermatitis. Lancet 2003;361:151–60. 12531593 10.1016/S0140-6736(03)12193-9

[7] Sidbury R Davis DM Cohen DE Guidelines of care for the management of atopic dermatitis: section 3. Management and treatment with phototherapy and systemic agents. J Am Acad Dermatol 2014;71:327–49. 24813298 10.1016/j.jaad.2014.03.030 PMC4410179

[8] Hengge UR Ruzicka T Schwartz RA Adverse effects of topical glucocorticosteroids. J Am Acad Dermatol 2006;54:1–5. 16384751 10.1016/j.jaad.2005.01.010

[9] Castellsague J Kuiper JG Pottegard A A cohort study on the risk of lymphoma and skin cancer in users of topical tacrolimus, pimecrolimus, and corticosteroids (Joint European Longitudinal Lymphoma and Skin Cancer Evaluation: JOELLE study). Clin Epidemiol 2018;10:299–310. 29559812 10.2147/CLEP.S146442 PMC5856050

[10] Denby KS Beck LA Update on systemic therapies for atopic dermatitis. Curr Opin Allergy Clin Immunol 2012;12:421–6. 22622476 10.1097/ACI.0b013e3283551da5

[11] Aubert-Wastiaux H Moret L Le Rhun A Topical corticosteroid phobia in atopic dermatitis: a study of its nature, origins and frequency. Br J Dermatol 2011;165:808–14. 21671892 10.1111/j.1365-2133.2011.10449.x

[12] Hon KL Ma KC Wong Y A survey of traditional Chinese medicine use in children with atopic dermatitis attending a paediatric dermatology clinic. J Dermatolog Treat 2005;16:154–7. 16096181 10.1080/09546630510038938

[13] Kaneko M Kishihara K Kawakita T Suppression of IgE production in mice treated with a traditional Chinese medicine, bu-zhong-yi-qi-tang (Japanese name: hochu-ekki-to). Immunopharmacology 1997;36:79–85. 9129999 10.1016/s0162-3109(96)00162-2

[14] Yamaoka Y Kawakita T Kishihara K Effect of a traditional Chinese medicine, bu-zhong-yi-qi-tang on the protection against an oral infection with Listeria monocytogenes. Immunopharmacology 1998;39:215–23. 9754907 10.1016/s0162-3109(98)00019-8

[15] Tahara E Effect of Kampo medicines on IgE-mediated biphasic skin reaction in mice. J Trad Med 1998;15:100–8.

[16] Kaneko M Kawakita T Nomoto K Inhibition of eosinophil infiltration into the mouse peritoneal cavity by a traditional Chinese medicine, bu-zhong-yi-qi-tang (Japanese name: Hochu-ekki-to). Immunopharmacol Immunotoxicol 1999;21:125–40. 10084334 10.3109/08923979909016398

[17] Ishimitsu R Nishimura H Kawauchi H Dichotomous effect of a traditional Japanese medicine, bu-zhong-yi-qi-tang on allergic asthma in mice. Int Immunopharmacol 2001;1:857–65. 11379041 10.1016/s1567-5769(01)00022-4

[18] Kobayashi H Mizuno N Kutsuna H Hochu-ekki-to suppresses development of dermatitis and elevation of serum IgE level in NC/Nga mice. Drugs Exp Clin Res 2003;29:81–4. 12951838

[19] Gao XK Fuseda K Shibata T Kampo medicines for mite antigen-induced allergic dermatitis in NC/Nga mice. Evid Based Complement Alternat Med 2005;2:191–9. 15937560 10.1093/ecam/neh077 PMC1142189

[20] Yanagihara S Kobayashi H Tamiya H Protective effect of Hochuekkito, a Kampo prescription, against ultraviolet B irradiation-induced skin damage in hairless mice. J Dermatol 2013;40:201–6. 23294358 10.1111/1346-8138.12050

[21] Kobayashi H Ishii M Tanii T Kampo therapies for atopic dermatitis: the effectiveness of Hochu-ekki-to. Nishinihon Hifu 1989;51:1003–13.

[22] Tsuji Y Tsujimoto Y Iikura Y Study of clinical efficacy of Hochu-ekki-to for child patients with atopic dermatitis. Rinsho Kenkyu 1993;70:4012–21.

[23] Kobayashi H Mizuno N Teramae H The effects of Hochu-ekki-to in patients with atopic dermatitis resistant to conventional treatment. Int J Tissue React 2004;26:113–7. 15648444

[24] Kobayashi H Ishii M Takeuchi S Efficacy and safety of a traditional herbal medicine, Hochu-ekki-to in the long-term management of Kikyo (delicate constitution) patients with atopic dermatitis: a 6-month, multicenter, double-blind, randomized, placebo-controlled study. Evid Based Complement Alternat Med 2010;7:367–73. 18955318 10.1093/ecam/nen003 PMC2887326

[25] Shamseer L Moher D Clarke M Preferred reporting items for systematic review and meta-analysis protocols (PRISMA-P) 2015: elaboration and explanation. BMJ 2015;350:g7647. 25555855 10.1136/bmj.g7647

[26] Moher D Liberati A Tetzlaff J Preferred reporting items for systematic reviews and meta-analyses: the PRISMA statement. PLoS Med 2009;6:e1000097. 19621072 10.1371/journal.pmed.1000097 PMC2707599

[27] Higgins JPT AD, Sterne JAC. Chapter 8: Assessing risk of bias in included studies. In: Higgins JPT Churchill R Chandler J Cumpston MS (editors), Cochrane Handbook for Systematic Reviews of Interventions version 5.2.0 (updated June 2017), Cochrane, 2017 Available at www.training.cochrane.org/handbook Accessed December 24, 2018

[28] Guyatt G Oxman AD Akl EA GRADE guidelines: 1. Introduction-GRADE evidence profiles and summary of findings tables. J Clin Epidemiol 2011;64:383–94. 21195583 10.1016/j.jclinepi.2010.04.026

[29] Deeks JJ HJ Altman DG Chapter 9: Analysing data and undertaking meta-analyses. In: Higgins JPT Churchill R Chandler J Cumpston MS (editors), Cochrane Handbook for Systematic Reviews of Interventions version 5.2.0 (updated June 2017), Cochrane, 2017 Available at www.training.cochrane.org/handbook Accessed December 24, 2018

[30] Sterne JAC EM Moher D Boutron I Chapter 10: Addressing reporting biases. In: Higgins JPT Churchill R Chandler J Cumpston MS (editors), Cochrane Handbook for Systematic Reviews of Interventions version 5.2.0 (updated June 2017), Cochrane, 2017 Available at: www.training.cochrane.org/handbook Accessed December 24, 2018

[31] Egger M Davey Smith G Schneider M Bias in meta-analysis detected by a simple, graphical test. BMJ 1997;315:629–34. 9310563 10.1136/bmj.315.7109.629 PMC2127453

[32] Bedi MK Shenefelt PD Herbal therapy in dermatology. Arch Dermatol 2002;138:232–42. 11843645 10.1001/archderm.138.2.232

[33] Gu S Yang AW Xue CC Chinese herbal medicine for atopic eczema. Cochrane Database Syst Rev. 2013 (9): Cd008642 10.1002/14651858.CD008642.pub2 PMC10639001 24018636

[34] Korea Institute of Oriental Medicine. Atopic dermatitis-Korean Medicine Clinical Practice Guideline. Seoul: ELSEVIER 2015

[35] Lee D Dong Yuan Ten Medical Books. Seoul: DS Printing Group; 1991.

[36] Dan K Akiyoshi H Munakata K A Kampo (traditional Japanese herbal) medicine, Hochuekkito, pretreatment in mice prevented influenza virus replication accompanied with GM-CSF expression and increase in several defensin mRNA levels. Pharmacology 2013;91:314–21. 23796966 10.1159/000350188

[37] Gou H Gu LY Shang BZ Protective effect of bu-zhong-yi-qi decoction, the water extract of Chinese traditional herbal medicine, on 5-fluorouracil-induced intestinal mucositis in mice. Hum Exp Toxicol 2016;35:1243–51. 26801985 10.1177/0960327115627686

[38] Zheng XF Tian JS Liu P Analysis of the restorative effect of bu-zhong-yi-qi-tang in the spleen-qi deficiency rat model using (1)H-NMR-based metabonomics. J Ethnopharmacol 2014;151:912–20. 24333365 10.1016/j.jep.2013.12.001

[39] Chinese Academy of Chinese Medical Sciences. Evidence-Based Guidelines of Clinical Practice in Chinese Medicine Specific Disease. Beijing: China Press of Traditional Chinese Medicine; 2011

[40] Chen D Fu X Fan R Expert consensus guidelines for diagnosis and treatment of atopic dermatitis with Chinese medicine. Chin J Dermatovenerol Integrated Tradit Western Med 2013;12:60–1.

