# Supplementary material for: The herbal drug, Bu-Zhong-Yi-Qi-Tang, for the treatment of atopic dermatitis: Protocol for a systematic review
Source: Medicine (Baltimore). 2019 Jan 4;98(1):e13938. doi: 10.1097/MD.0000000000013938 (PMC6344144; doi:10.1097/MD.0000000000013938)
Supplement: Supplemental Digital Content [file medi-98-e13938-s001.docx]

**Appendix 1. Search Strategy**

**PubMed search strategy**

1. Atopic dermatitis [MeSH Terms]
2. Eczema [MeSH Terms]
3. Neurodermatitis [Title/Abstract]
4. Dermatitis [Title/Abstract]
5. #1 or 2-4
6. Herbal medicine [MeSH Terms]
7. Chinese herbal drugs [MeSH Terms]
8. Medicine, korean traditional [MeSH Terms]
9. Medicine, kampo [MeSH Terms]
10. Traditional Chinese medicine [MeSH Terms]
11. Plant extracts [Title/abstract]
12. Bojungikki-tang
13. Bu-Zhong-Yi-Qi-Tang
14. Hochu-ekki-to
15. #6 or 7-14
16. #5 and 15

**CNKI search strategy**

(SU=('异位性皮炎'+'异位性湿疹'+'特应性皮炎'+'特异性湿疹'+'婴幼儿湿疹'+'儿童湿疹') or TI=('异位性皮炎'+'异位性湿疹'+'特应性皮炎'+'特异性湿疹'+'婴幼儿湿疹'+'儿童湿疹') or KY=('异位性皮炎'+'异位性湿疹'+'特应性皮炎'+'特异性湿疹'+'婴幼儿湿疹'+'儿童湿疹') or AB=('异位性皮炎'+'异位性湿疹'+'特应性皮炎'+'特异性湿疹'+'婴幼儿湿疹'+'儿童湿疹') or FT=('异位性皮炎'+'异位性湿疹'+'特应性皮炎'+'特异性湿疹'+'婴幼儿湿疹'+'儿童湿疹')) and (SU=('补中益气汤'+'补中益气'+ 'BuZhongYiQiTang'+'BuZhongYiQi') or TI=('补中益气汤'+'补中益气'+ 'BuZhongYiQiTang'+'BuZhongYiQi') or KY=('补中益气汤'+'补中益气'+ 'BuZhongYiQiTang'+'BuZhongYiQi') or AB=('补中益气汤'+'补中益气'+ 'BuZhongYiQiTang'+'BuZhongYiQi') or FT=('补中益气汤'+'补中益气'+ 'BuZhongYiQiTang'+'BuZhongYiQi'))
